# Improved prediction of post-translational modification crosstalk within proteins using DeepPCT

**DOI:** 10.1093/bioinformatics/btae675

**Published:** 2024-11-21

**Authors:** Yu-Xiang Huang, Rong Liu

**Affiliations:** Hubei Key Laboratory of Agricultural Bioinformatics, College of Informatics, Huazhong Agricultural University, Wuhan 430070, P.R. China; Hubei Key Laboratory of Agricultural Bioinformatics, College of Informatics, Huazhong Agricultural University, Wuhan 430070, P.R. China

## Abstract

**Motivation:**

Post-translational modification (PTM) crosstalk events play critical roles in biological processes. Several machine learning methods have been developed to identify PTM crosstalk within proteins, but the accuracy is still far from satisfactory. Recent breakthroughs in deep learning and protein structure prediction could provide a potential solution to this issue.

**Results:**

We proposed DeepPCT, a deep learning algorithm to identify PTM crosstalk using AlphaFold2-based structures. In this algorithm, one deep learning classifier was constructed for sequence-based prediction by combining the residue and residue pair embeddings with cross-attention techniques, while the other classifier was established for structure-based prediction by integrating the structural embedding and a graph neural network. Meanwhile, a machine learning classifier was developed using novel structural descriptors and a random forest model to complement the structural deep learning classifier. By integrating the three classifiers, DeepPCT outperformed existing algorithms in different evaluation scenarios and showed better generalizability on new data owing to its less distance dependency.

**Availability and implementation:**

Datasets, codes, and models of DeepPCT are freely accessible at https://github.com/hzau-liulab/DeepPCT/.

## 1 Introduction

Post-translational modifications (PTMs) widely exist in eukaryotic proteins and play a critical role in many biological processes, such as signal transduction, cellular localization, and immune response (Liu *et al.* 2016, Chen *et al.* 2020). PTM-induced regulations refer to not only the effect of individual modification sites but also the combined influence of multiple modification sites. For instance, phosphorylation of Ser10 in histone H3 could impair the binding of effector proteins to methylated Lys9, thereby leading to different functional outcomes, which is known as the ‘methyl/phos switch’ model (Fischle *et al.* 2003). This dynamic interplay between PTMs within the same protein or across different interacting proteins is termed PTM crosstalk (Venne *et al.* 2014). Accurate identification of PTM crosstalk events could help to elucidate the complicated regulatory mechanism, providing a foundation for investigating protein functions. Although mass spectrometry techniques have been commonly used for the high-throughput detection of PTM sites, the large-scale experimental identification of PTM crosstalk remains a considerable challenge (Witze *et al.* 2007, Adoni *et al.* 2022). Accordingly, there is a pressing need to develop computational approaches to predict crosstalk events.

During the past two decades, a series of computational studies have been devoted to the characterization and identification of PTM crosstalk. Beltrao *et al.* discovered that the functional importance of PTMs is associated with their conservation, which could be used to identify regulatory regions within protein families (Beltrao *et al.* 2013). Schweiger *et al.* found that cooperative phosphorylation sites form clusters in the protein sequence, while Korkuc *et al.* showed that these sites are spatially adjacent to each other (Schweiger and Linial 2010, Korkuc and Walther 2016, 2017). Woodsmith *et al.* (2013) and Pejaver *et al.* (2014) revealed that PTM-enriched sequence segments are highly related to intrinsically disordered regions. Based on these findings, Huang *et al.* (2015) developed PTM-X, which is the first machine learning algorithm to predict PTM crosstalk within proteins. They used the distance measures, co-evolutionary signals, and co-localization attributes of PTM pairs as the input of a naive Bayes classifier. PTM-X strongly depended on residue pair-based features and sequence information. Five years ago, we developed the PCTpred method to explore the utility of residue-based features and structural information (Liu and Liu 2020). This model included a structural classifier and a sequence classifier, both of which were implemented by combining a group of residue and residue pair-based features with random forest algorithms. Recently, Deng *et al.* (2023) designed a structure-based algorithm by incorporating protein dynamic features. Moreover, they explored convolutional neural networks and long-short term memory networks to predict PTM crosstalk, but the deep learning model performed worse than the random forest model.

Despite the advancements achieved by existing studies, several problems could be further explored. First, the application of deep learning techniques to predict PTM crosstalk remains challenging due to the scarcity of experimentally validated data. It is highly needed to design suitable deep leaning strategies to learn complex patterns and correlations from very limited training data. Second, incorporation of structural features could enhance the performance, but the structural information of numerous samples is unavailable. With the recent advances in protein structure prediction (e.g. AlphaFold series) (Jumper *et al.* 2021), the high-confidence predicted structures could be used to substitute native counterparts. Third, existing methods only adopted hand-crafted sequence and structural features in this prediction task. The embedding generated from pretrained models exhibited better performance in various functional prediction problems and could thus be extended to the identification of PTM crosstalk (Jiang *et al.* 2023, Zeng *et al.* 2023, Kulmanov *et al.* 2024). Fourth, all the previous studies lacked an independent testing. Especially, PCTpred and Deng *et al.*’s model were optimized on the whole data using the feature selection algorithm, which could result in overfitting issues (Liu and Liu 2020, Deng *et al.* 2023). The proposed model should be rigorously assessed using external data to ensure its generalizability.

Inspired by the above problems, we present a novel deep learning framework, named DeepPCT, to identify PTM crosstalk events within proteins. In this algorithm, one deep learning classifier was built for sequence-based prediction by combining the residue and residue pair embeddings with cross-attention techniques, while the other classifier was established for structure-based prediction by integrating the structural embedding and a graph neural network. Meanwhile, a machine learning classifier was established using a series of novel structural descriptors and a random forest model to complement the structural deep learning classifier. By integrating the three classifiers, DeepPCT not only outperformed existing methods for both sample- and protein-based evaluation but also showed better generalizability on new test data due to its less distance dependency. Especially, the inference time of DeepPCT was 60 times faster than that of our previous model.

## 2 Materials and methods

### 2.1 Data collection

In this study, the experimentally identified PTM crosstalk pairs within proteins were collected from previous works and relevant literatures. The dataset of our earlier study, which contained 205 crosstalk pairs and 12,468 negative samples from 81 proteins, was used for model training in the current study. The 205 positive samples, including 193 pairs collected by Huang *et al.* and 12 pairs collected by our group, were reported in the literature prior to September 2017 (Huang *et al.* 2015, Liu and Liu 2020). Moreover, we used the keyword combination ‘(cross AND (talk OR regulate OR link)) OR interplay OR interaction) AND post translational modification’ to retrieve the references between September 2017 and April 2023 from the PubMed database. By reading the resultant 898 articles, we additionally obtained 20 crosstalk pairs from 9 human proteins (Supplementary Table S1). Following the previous studies, the PTM sites detected by low-throughout experiments in the PhosphoSitePlus database were used to generate negative samples (Hornbeck *et al.* 2015). We generated all possible PTM pairs within each protein and deleted pairs whose both sites were included in the positive set, resulting in 427 negative samples. The newly collected samples were used for independent testing. To investigate the impact of distances between PTM sites on prediction performance, we prepared another three datasets (i.e. Set-random, Seq-control, and Str-control), in which all the positive samples and an equal number of negative samples were chosen from the training set. For Set-random, the negative samples were randomly selected based on the native distance distribution. For Seq-control and Str-control, the negative samples were chosen based on the distributions of sequence and structural distances of positive samples, respectively (Supplementary Text S1). For the aforementioned 90 proteins, the predicted structures were used for training and testing. The structures generated by AlphaFold2 were downloaded from AlphaFold DB (Varadi *et al.* 2022).

### 2.2 Overview of DeepPCT

**Figure 1.**
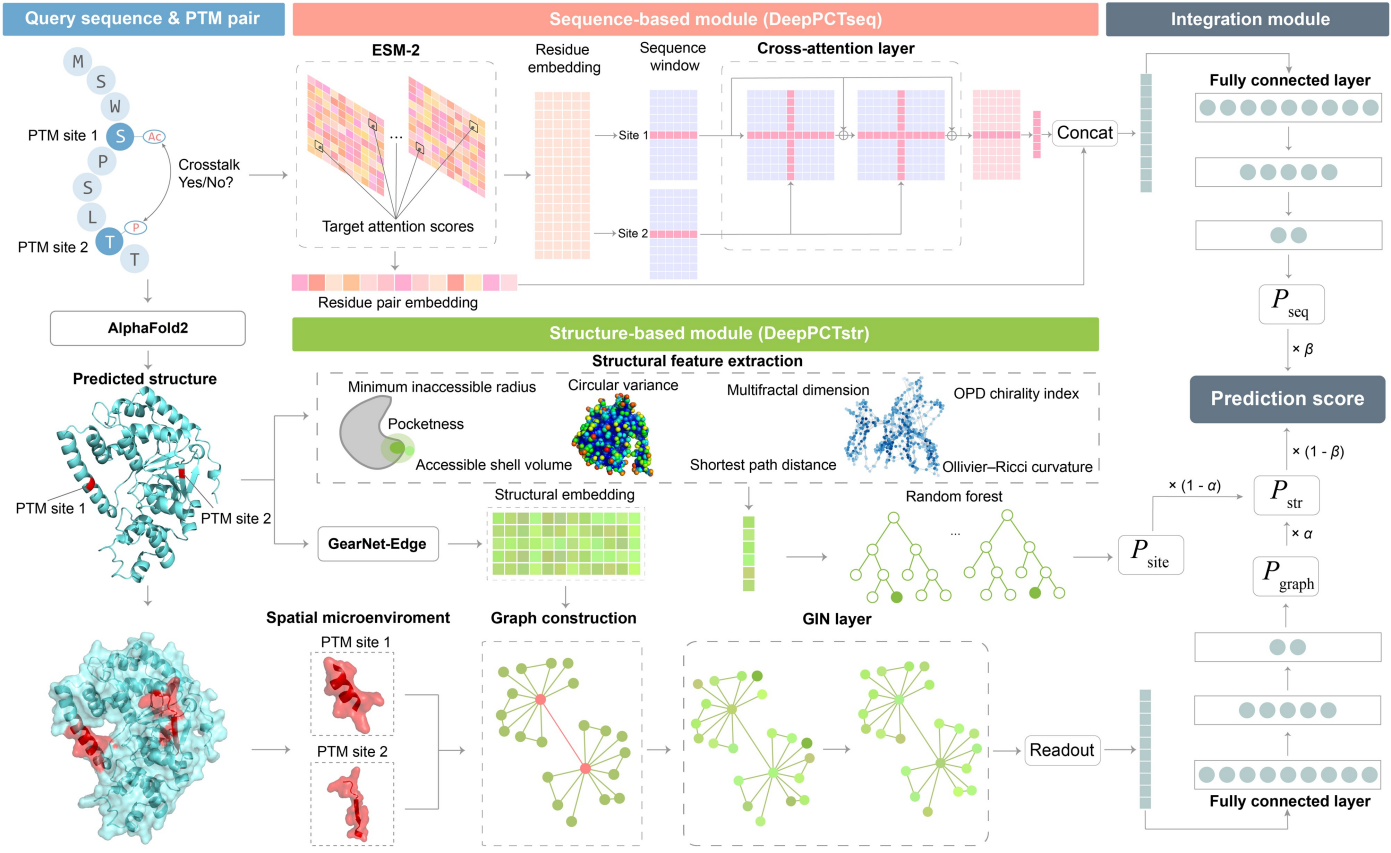
Flowchart of DeepPCT for PTM crosstalk prediction. Our algorithm includes a sequence-based module (DeepPCTseq), a structure-based module (DeepPCTstr), and an integration module. DeepPCTseq combines the residue and residue pair embeddings with cross-attention techniques to predict crosstalk events. DeepPCTstr comprises two submodules, namely DeepPCTgraph and DeepPCTsite. The former is dependent on the structural embedding and a graph neural network, and the latter is based on a group of novel structural descriptors and a random forest model. The integration module is the weighted sum of the outputs from the above three classifiers.

As shown in Fig. 1, the DeepPCT algorithm is composed of a sequence-based module (DeepPCTseq), a structure-based module (DeepPCTstr), and an integration module. For a query PTM pair, the structure of the involving protein was predicted by AlphaFold2 (Jumper *et al.* 2021). Sequence- and structure-based predictions were then conducted using DeepPCTseq and DeepPCTstr, respectively. Regarding the sequence component, the representations of individual residues and relevant residue pairs were generated by a pretrained protein language model to capture the sequential pattern and association of two PTM sites. The residue embedding was processed by a cross-attention model and then combined with the residue pair embedding to calculate the crosstalk probability using a fully connected layer. As for the structural section, DeepPCTstr included two submodules: DeepPCTgraph and DeepPCTsite. The former generated the structural embedding of each residue through a pretrained protein structural model. A graph neural network was then applied to the graph representations of each PTM pair for evaluating the crosstalk probability. Meanwhile, the latter generated comprehensive structural descriptors to describe two PTM sites and adopted random forests to assess their crosstalk probability (Breiman 2001). To utilize the interplay of three classifiers, their outputs were integrated to generate the final prediction score using a weighted combination strategy.

### 2.3 Feature extraction

#### 2.3.1 Sequence-based features

##### 2.3.1.1 Residue embedding

Trained on a large number of protein sequences through the unsupervised learning approach, protein language models (PLMs) could effectively capture the latent information within protein sequences. In this study, we adopted the ESM-2 for residue encoding by comparing different PLMs (Supplementary Text S2 and Table S2). The representations from the last layer of this model were extracted as residue embedding (Lin *et al.* 2023).

##### 2.3.1.2 Residue pair embedding

ESM-2 was also used to generate embedding features for residue pairs. This model comprises 33 transformer encoder layers, each of which contains 20 attention heads in the multi-head attention block. To generate residue pair embedding, the attention map (i.e. $\text{Softmax}\left({QK}^{T}/\sqrt{d_{k}}\right)$ in the attention formula) was extracted from each attention head. For a sequence, a total of 660 (33 × 20) attention maps could be generated. The attention matrix ***A*** has a dimension of L×L, where *L* represents the sequence length. Each attention score in the matrix evaluates the dependency between two residues, which could be a potential indicator of related PTM sites. For a given sample $\left(i,j\right)$, because A is asymmetric, two attention scores, $A_{ij}$ and $A_{ji}$, should be extracted. Finally, the attention scores from the 660 attention maps constituted a 1320-dimensional vector for each pair.

#### 2.3.2 Structure-based features

##### 2.3.2.1 Structural embedding

The structural representations of residues could also be generated by pre-trained protein models. Here, we employed the GearNet-Edge model to convert a protein structure into the graph representation of residues (nodes) (Zhang *et al.* 2023). The node features derived from the resulting graph were used as structural embeddings to predict PTM crosstalk.

##### 2.3.2.2 Structural descriptors

A group of geometry- and graph-based descriptors were adopted to characterize the structural context of each residue that may not be fully represented by structural embeddings. Among the geometry-based descriptors, the circular variance reflects the density of neighboring atoms of a residue. The Osipov-Pickup-Dunmur (OPD) chirality index illustrates the stereochemical property of a residue’s local context and could serve as an indicator of secondary structure. Besides, three local geometric descriptors, including the accessible shell volume, minimum inaccessible radius, and pocketness, quantify the depth and exposure of residues. The graph-based descriptors were used to depict the topological properties of residues in a network. The Ollivier-Ricci curvature measures the geodesic path complexity of a residue based on Riemannian geometry, while the multifractal dimension measures the network complexity in multifractal analysis. The shortest path distance could quantify the spatial proximity of two residues. Detailed information about these features is provided in Supplementary Text S3 and Table S3.

### 2.4 Model construction

#### 2.4.1 Sequence-based module

For a PTM pair $\left(i,j\right)$, this module took two sequence windows, namely $X^{\left(i\right)}$ and $X^{\left(j\right)}$, along with a residue pair embedding $X^{\left(ij\right)}$ as input. Each sequence window was a matrix composed of residue embeddings of 11 consecutive residues centered on the PTM site. Zero-padding was used to represent the missing residues if the target site appeared at both ends of a sequence. A two-layer cross-attention network was adopted to convert the two sequence windows into a merged representation (Vaswani *et al.* 2017). Specifically, a residual connection followed by layer normalization was applied to each network layer as follows (He *et al.* 2016):

$$H^{\left(l\right)}=\text{LayerNorm}\left(f^{\left(l\right)}\left(H^{\left(l-1\right)}\right)+H^{\left(l-1\right)}\right)$$

where $H^{\left(l\right)}$ denotes the output from the lth layer, and $H^{\left(0\right)}$ is the initial input $X^{\left(i\right)}$. In the layer function $f^{\left(l\right)}$, five parallel attention heads were used to depict interactions between sequence windows. To this end, $H^{\left(l-1\right)}$ was transformed into the query ($Q^{\left(i\right)}$), while $X^{\left(j\right)}$ was transformed into the key ($K^{\left(j\right)}$) and value ($V^{\left(j\right)}$):

$$Q^{\left(i\right)},K^{\left(j\right)},V^{\left(j\right)}=H^{\left(l-1\right)}W^{Q}+b^{Q},X^{\left(j\right)}W^{K}+b^{K},X^{\left(j\right)}W^{V}+b^{V}$$

$$H_{m}^{\left(l\right)}=\text{Softmax}\left(\frac{Q^{\left(i\right)}{\left(K^{\left(j\right)}\right)}^{T}}{\sqrt{d}}\right)V^{\left(j\right)},m=1,2,\ldots ,5$$

where $W^{Q}$, $W^{K}$, and $W^{V}$ denote learnable weight matrices, and $b^{Q}$, $b^{K}$, and $b^{V}$ are biases. The scaling factor d was set to 256. $H_{m}^{\left(l\right)}$ represents the output of the mth attention head in the lth layer. The outputs of these attention heads were merged as follows:

$$f^{\left(l\right)}\left(H^{\left(l-1\right)}\right)≔\text{concatenate}\left(H_{1}^{\left(l\right)},H_{2}^{\left(l\right)},\ldots ,H_{5}^{\left(l\right)}\right)W^{O}+b^{O}$$

where $W^{O}$ is a projection matrix, and $b^{O}$ is a learnable bias. The final layer’s output $H^{\left(2\right)}$ contained 11 vectors and was the fused representation of two sequence windows. The central vector $h_{1}=H_{6,\cdot }^{\left(2\right)}$ was combined with the pair embedding $X^{\left(ij\right)}$ to form a concatenated vector $h_{2}$, which was then fed into a three-layer fully connected network to yield the output probability as follows:

$$P_{\text{seq}}=\text{Softmax}\left(\text{LeakyReLU}\left(\text{BatchNorm}\left(h_{2}W_{1}+b_{1}\right)\right)W_{2}+b_{2}\right)$$

where $W_{1}$, $W_{2}$, $b_{1}$, and $b_{2}$ are learnable parameters. We implemented this module using PyTorch (Paszke *et al.* 2019). Early stopping was adopted if a notable decrease in performance was observed. The cross-entropy loss was used for loss calculation, and the parameters were updated using the Adam optimizer with a learning rate of 1e−5 (Kingma and Ba 2014).

#### 2.4.2 Structure-based module

##### 2.4.2.1 Graph-based submodule

To develop this submodule, we extracted the spatial microenvironment S of each PTM site, which included the target residue and its 10 nearest residues in terms of structural distances. For a PTM pair $\left(i,j\right)$, as illustrated in Fig. 1, their microenvironments were converted into a graph representation $G=\left(V,E\right)$, where each node denotes a residue, and an edge is formed between (1) PTM sites i and j; (2) PTM site i (or j) and residues in its microenvironment $S^{\left(i\right)}$ (or $S^{\left(j\right)}$); (3) two residues within $S^{\left(i\right)}$ (or $S^{\left(j\right)}$) that are chemically interconnected. The structural embedding treated with layer normalization was adopted as the node feature. This graph was fed into a two-layer graph isomorphism network (GIN) for updating its node feature (Xu *et al.* 2019). Let $h_{v}^{\left(0\right)}$ be the initial feature of node v∈V, and the updating process can be presented as follows:

$$h_{v}^{\left(l\right)}=f_{\Theta }^{\left(l\right)}\left(h_{v}^{\left(l-1\right)}+\underset{u\in N\left(v\right)}{\max}\left\{h_{u}^{\left(l-1\right)}\right\}\right)$$

$$f_{\Theta }^{\left(l\right)}\left(X\right)≔XW^{\left(l\right)}+b^{\left(l\right)}$$

where $N\left(v\right)$ is a set of nodes adjacent to v, and $f_{\Theta }^{\left(l\right)}$ is the apply function in the lth layer, which contains the learnable parameters $W^{\left(l\right)}$ and $b^{\left(l\right)}$. Subsequently, a readout function was used to obtain the graph representation $H_{G}$ as follows:

$$H_{G}=\text{readout}\left(\left\{h_{v}^{\left(2\right)}|v\in V\right\}\right)≔\text{concatenate}\left(\left\{h_{v}^{\left(2\right)}|v\in V\right\}\right)W^{\prime }$$

where $W^{\prime }$ is a learnable projection matrix. Finally, $H_{G}$ was input into a linear layer to generate a prediction probability as follows:

$$P_{\text{graph}}=\text{Softmax}\left(\text{LeakyReLU}\left(H_{G}\right)W^{\prime \prime }+b^{\prime \prime }\right)$$

where $W^{\prime \prime }$ and $b^{\prime \prime }$ are learnable parameters. This model was implemented using the DGL and PyTorch (Paszke *et al.* 2019, Wang *et al.* 2019). Early stopping was used in the training process. The cross-entropy loss was used as the loss function. To avoid overfitting, we incorporated the flooding technique into our loss function, in which the flood level (b) was set to 0.017 (Ishida *et al.* 2020). The Adam optimizer with a learning rate of 5e−6 was adopted for model optimization (Kingma and Ba 2014).

##### 2.4.2.2 Site-based submodule

This submodule used hand-crafted structural descriptors as the input of random forest algorithms (Breiman 2001). Among these descriptors, the shortest path distance was a residue pair-based descriptor, and the remaining features were residue-based descriptors. The maximum, minimum and average values of both PTM sites were computed for residue-based descriptors. We adopted the scikit-learn package to implement this model with a configuration of 500 trees (Pedregosa *et al.* 2011).

#### 2.4.3 Integration module

We used a weighted combination method to integrate the results of different modules. Specifically, the output probability of the structure-based module ($P_{\text{str}}$) was the weighted sum of probabilities from the graph- and site-based submodules ($P_{\text{graph}}$ and $P_{\text{site}}$, respectively). The output probability of our final model (P) was the weighted sum of probabilities from the sequence- and structure-based modules ($P_{\text{seq}}$ and $P_{\text{str}}$, respectively). The formulas are shown as follows:

$$P_{\text{str}}=\alpha P_{\text{graph}}+\left(1-\alpha \right)P_{\text{site}}$$

$$P=\beta P_{\text{seq}}+\left(1-\beta \right)P_{\text{str}}$$

where α and β are the weight factors. Due to the imbalanced ratio between positive and negative samples, the cutoff of our method is relatively small. The optimal values for α, β, and the cutoff are 0.65, 0.65, and 0.15, respectively (Supplementary Fig. S1).

### 2.5 Performance evaluation

To evaluate our algorithm, we conducted 10-fold cross-validation (CV) on the training set and independent testing on the newly collected samples. When performing 10-fold CV, we adopted sample- and protein-based evaluations. The former randomly divided all samples into different subsets for training and validation, and the latter allocated proteins into the subsets to ensure that test samples were derived from the unseen proteins in the training process. The hyperparameters of our algorithm were determined by protein-based evaluations, because this strategy is more rigorous and closer to the real application. To train deep learning models, we used all the positive and negative samples in the training set. Seven widely used measures, including the area under the receiver operating characteristic (ROC) curve (AUC), area under the precision-recall curve (AUPR), Matthew’s correlation coefficient (MCC), F1-score, recall, precision, and accuracy (ACC), were used for assessing the performance of our model. We also evaluated significant differences in performance between different models. For a given dataset, we randomly chosen 70% of the crosstalk and negative pairs 10 times and calculated AUC and AUPR values. The Anderson-Darling test was then used to assess whether these values obey a normal distribution. Based on the normality assumption, the paired *t*-test or Wilcoxon rank-sum test was selected for statistical testing.

## 3 Results

### 3.1 Effectiveness of three basic classifiers

We evaluated the performance of three basic classifiers on the training set using 10-fold CV. As shown in Table 1, DeepPCTseq performed most favorably for sample-based evaluation, with an AUC and AUPR of 0.946 and 0.643, and outperformed two structure-based classifiers (i.e. DeepPCTsite and DeepPCTgraph) by approximately 0.05 and 0.04 in AUC and 0.3 and 0.2 in AUPR, respectively. Regarding the more rigorous protein-based evaluation, we observed a notable decrease in the performance of three classifiers. Even so, the sequence-based model still surpassed structure-based counterparts. DeepPCTsite and DeepPCTgraph achieved a more remarkable decrease in AUC from 0.894 and 0.911 to 0.736 and 0.739, respectively, while the measure of DeepPCTseq decreased from 0.946 to 0.827. This suggests that sequence information is more crucial and exhibits greater robustness in predicting PTM crosstalk compared to structural information.

**Table 1.** Performance of different modules on training set using sample- and protein-based evaluation.

Classifier	Sample-based evaluation				Protein-based evaluation			
	AUC	AUPR	MCC	F1	AUC	AUPR	MCC	F1
DeepPCTsite	0.894	0.302	0.338	0.280	0.736	0.149	0.138	0.115
DeepPCTsite^AF3^	0.889	0.290	0.344	0.336	0.744	0.163	0.174	0.193
DeepPCTgraph	0.911	0.493	0.476	0.435	0.739	0.182	0.167	0.183
DeepPCTgraph^AF3^	0.924	0.538	0.548	0.526	0.747	0.189	0.179	0.158
DeepPCTstr	0.942	0.525	0.503	0.471	0.786	0.217	0.211	0.228
DeepPCTstr^AF3^	0.947	0.572	0.557	0.553	0.799	0.224	0.222	0.242
DeepPCTseq^nrp^	0.925	0.586	0.569	0.556	0.775	0.269	0.222	0.215
DeepPCTseq	0.946	0.643	0.596	0.587	0.827	0.368	0.318	0.271
DeepPCT	0.957	0.663	0.620	0.616	0.834	0.375	0.349	0.337
DeepPCT^AF3^	0.956	0.662	0.627	0.627	0.838	0.381	0.357	0.348

AF3 denotes that AlphaFold3-based predicted structures were used.

DeepPCTseq^nrp^ denotes that the residue pair embedding was deleted in DeepPCTseq.

Meanwhile, we constructed baseline models for each basic classifier (Supplementary Text S4). Relative to our two deep learning classifiers (i.e. DeepPCTseq and DeepPCTgraph), we established baselines using the convolutional neural network (CNN) and long-short term memory network (LSTM). For the graph-based classifier, we constructed three additional baselines using graph convolutional network (GCN), graph attention network (GAT), and GraphSAGE. For the machine learning classifier (i.e. DeepPCTsite), we additionally evaluated five commonly used machine learning algorithms. As shown in Supplementary Table S4, all the baseline models were inferior to the finally selected models. Notably, the CNN and LSTM baselines for DeepPCTgraph yielded relatively worse results, whereas the graph-based baselines generated a slightly declined performance. This is probably because the CNN and LSTM models cannot handle the graph information, suggesting the importance of constructing graph representations for PTM pairs in our approach.

**Figure 2.**
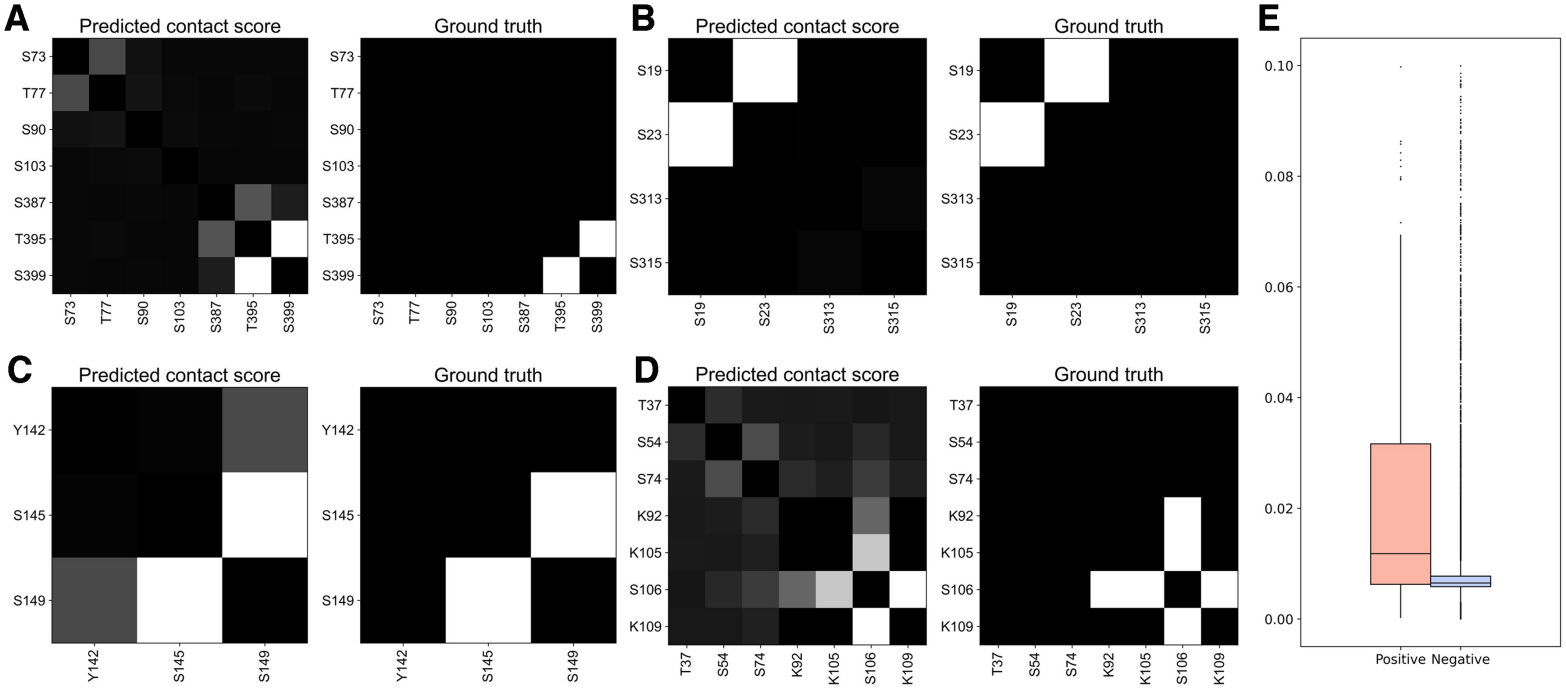
Comparison of predicted contact scores of crosstalk and negative pairs based on residue pair embeddings. (A-D) Visualization of predicted contact score and ground truth for representative examples. UniProt IDs: (A) P24864, (B) Q15653, (C) Q9UKT4, and (D) Q99741. (E) Distribution of predicted contact scores for crosstalk and negative pairs.

**Figure 3.**
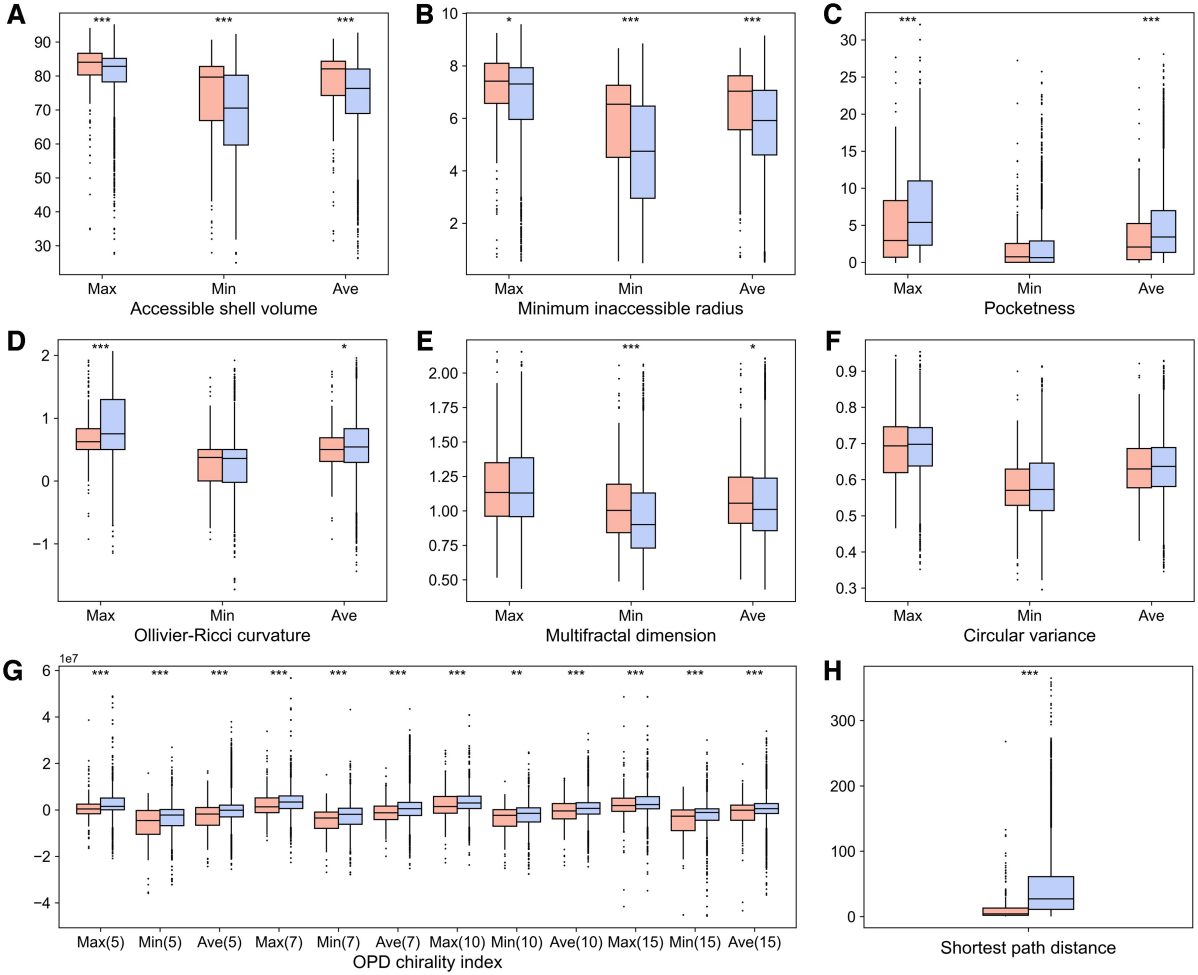
Comparison of structural features between crosstalk (red) and negative pairs (blue). (A) Accessible shell volume. (B) Minimum inaccessible radius. (C) Pocketness. (D) Ollivier-Ricci curvature. (E) Multifractal dimension. (F) Circular variance. (G) OPD chirality index (calculated using *N* = 5, 7, 10, and 15). (H) Shortest path distance. Max, Min, and Ave represent the maximum, minimum, and average values of two residues in each PTM pair regarding a given attribute. The statistical significance is evaluated using the Wilcoxon rank sum test. ***P < 0.001, **P < 0.01, and *P < 0.05.

Additionally, we explored the interpretability of representative features adopted in each classifier. Recently, Rao *et al.* demonstrated that the attention scores from PLMs can accurately predict residue contacts in proteins through a simple logistic regression method (Rao *et al.* 2020). Inspired by this work, we utilized the residue pair embedding to characterize the association between PTM sites for sequence-based prediction. To analyze this feature, we applied their method to transform the embeddings into single contact scores and compared the scores of positive and negative samples. It is clear that the contact scores of crosstalk pairs were generally higher in relevant proteins (Fig. 2A−D), and there was a significant difference between all the positive and negative samples (Fig. 2E, *P* = 1.97e−28), implying the potential of the residue pair embedding to identify crosstalk pairs. As shown in Table 1, excluding this embedding resulted in a remarkable decrease in the performance of sequence-based classifiers (DeepPCTseq^nrp^ versus DeepPCTseq). For the structure-based prediction, a group of hand-crafted descriptors were newly introduced to complement the structural embedding. We found that crosstalk pairs tended to be located on the protein surface (Fig. 3A, B, and F), be within the shallower region of protein pockets (Fig. 3C), and be spatially proximate in protein structures (Fig. 3H). This may be because residues that are deeply buried or quite distant from each other could hinder their interactions. Moreover, the OPD chirality indices of crosstalk pairs were significantly lower than those of negative samples (Fig. 3G). This measure could be correlated with the secondary structure status of a residue (Supplementary Fig. S2). Namely, the crosstalk pairs were enriched in the coil region, where residues generally have smaller chirality index values. The above results suggest these features could capture certain patterns to distinguish crosstalk pairs from negative pairs.

### 3.2 Integration of basic classifiers improves prediction performance

**Figure 4.**
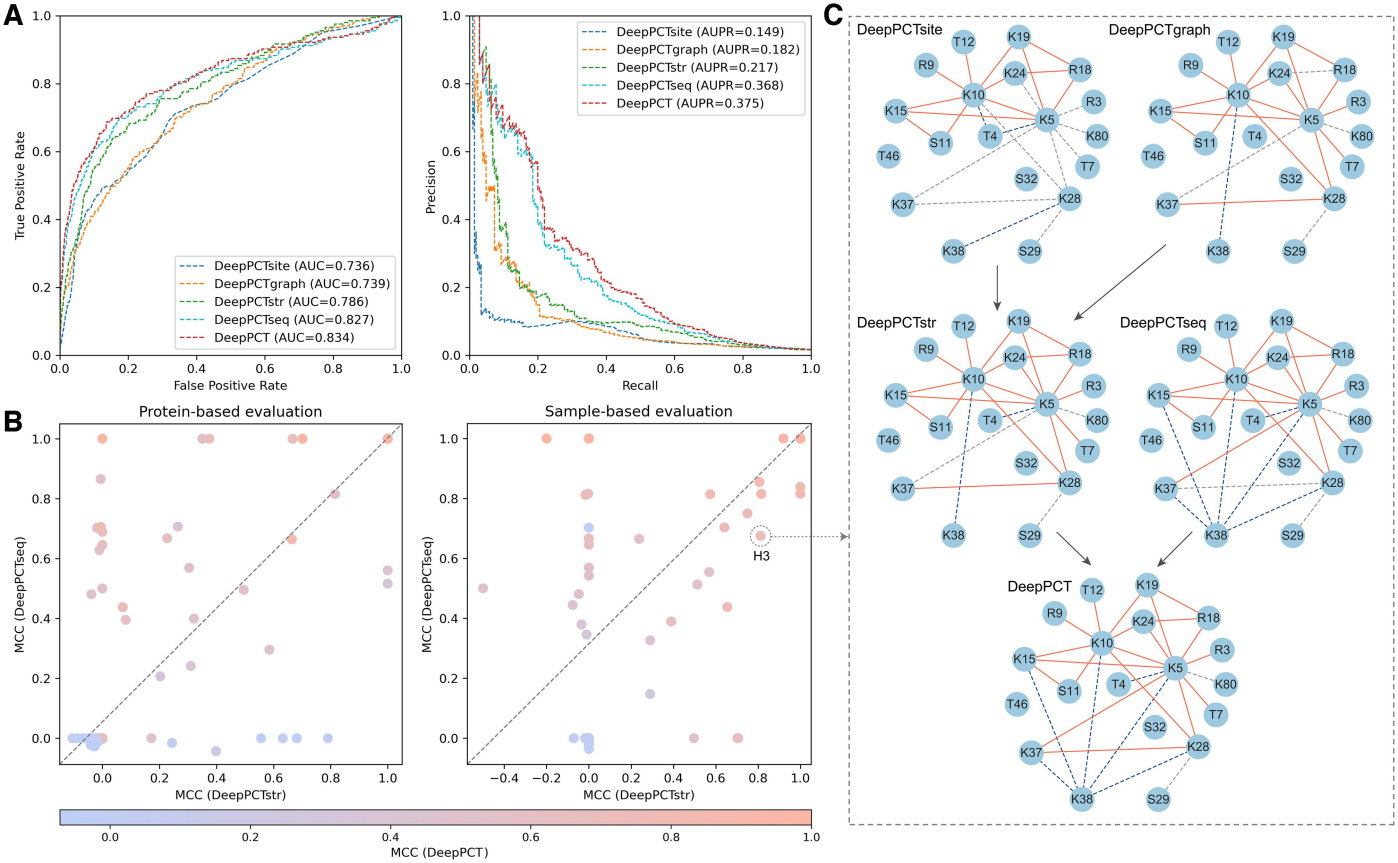
Comparative analysis of three basic classifiers and their integrations. (A) ROC (left) and precision-recall (right) curves for DeepPCT and its modules on training set. (B) Scatter plots of MCC on training set using protein- and sample-based evaluations. Each point denotes the MCC value of all samples in a given protein. (C) Visualization of prediction results of PTM pairs in histone H3 using sample-based evaluation. True positives, false positives, and false negatives are highlighted in red, blue, and gray, respectively.

To explore the complementarity among three basic classifiers, we evaluated the performance of integrated modules (i.e. DeepPCTstr and DeepPCT) on the training set. As shown in Fig. 4A and Table 1, although DeepPCTsite achieved the lowest measures among basic classifiers, it provided a complement to the other structure-based classifier. Through combining DeepPCTgraph with DeepPCTsite, even for the stringent protein-based evaluation, the AUC and AUPR values were improved from 0.739 and 0.182 to 0.786 and 0.217, respectively. This suggests that the hand-crafted descriptors effectively complement the embedding features in capturing the structural patterns of crosstalk sites. When we further integrated DeepPCTseq with DeepPCTstr, the AUC and AUPR values increased from 0.827 and 0.368 to 0.834 and 0.375, respectively. Figure 4B illustrates that both sequence- and structure-based modules achieved better MCC values for partial proteins. The Pearson correlation coefficients between their results were 0.406 and 0.570 for protein- and sample-based evaluations, respectively, implying that these modules could complement each other. For instance, the number of true positives in the histone H3 gradually increased with the integration of various classifiers in the sample-based evaluation (Fig. 4C). Finally, DeepPCT successfully identified 20 pairs among the 22 positive samples, while yielding 6 false positives out of 34 negative samples. For the protein-based evaluation, nevertheless, DeepPCTseq performed poorly on this protein, generating only three true positives (Supplementary Fig. S3). After including DeepPCTstr, the number of true positives increased from three to nine (DeepPCTseq versus DeepPCT), while that of false positives decreased from five to one (DeepPCTstr versus DeepPCT). This further indicates that the sequence- and structure-based modules could provide different information. Besides, we tried different strategies to integrate the sequence- and structure-based information into a deep learning framework, but these attempts failed to surpass the weighted combination approach (Supplementary Text S5 and Table S5).

### 3.3 DeepPCT exhibits less distance dependency than other methods

**Figure 5.**
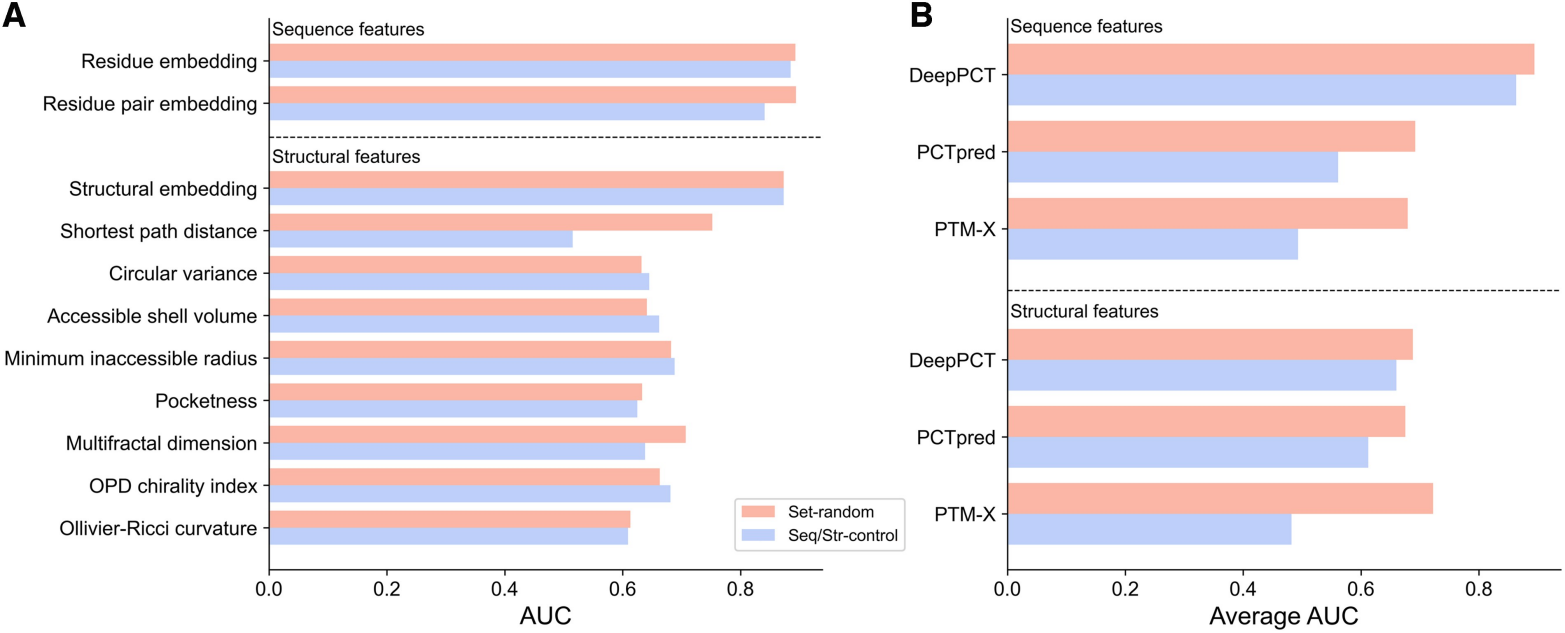
Analysis of distance dependency of different features and methods. (A) Performance of individual features of DeepPCT on Set-random and Seq/Str-control. (B) Average performance of sequence and structural features used by DeepPCT and other methods on Set-random and Seq/Str-control.

PTM sites involved in crosstalk events are generally proximate to each other at both the sequence and structural levels. Our previous study has indicated that the residue pair-based attributes (e.g. distance, co-evolution, and co-localization), which strongly depend on the distance between PTMs, might have difficulty in identifying remote crosstalk sites and adjacent unrelated sites (Liu and Liu 2020). To investigate this dependency, here, we built a random forest predictor for each feature used by our and other studies and assessed the performance on three datasets, including a randomly sampled dataset (i.e. Set-random) and two distance-constrained datasets (i.e. Seq-control and Str-control). Only the shortest path distance exhibited a remarkable performance decrease on distance-constrained samples in the present method (Fig. 5A). Additionally, the descriptors used by DeepPCT yielded a much less average loss than those used by existing methods (Fig. 5B). Among the features adopted in this work, the residue embedding, residue pair embedding, and structural embedding obtained the highest performance on Set-random, with an AUC of approximately 0.880, while the AUCs of other descriptors ranged from 0.613 to 0.752. Particularly, the residue and structural embeddings achieved robust results on distance-constrained datasets, with a decrease of approximately 0.008 and 0.001 in AUC, respectively, compared with a value of approximately 0.05 for the residue pair embedding. The remarkable decline could be due to the following reasons. First, the sequence distance could be implicitly involved in this feature during the positional encoding of the ESM-2 model (Lin *et al.* 2023). Second, the attention scores of PLMs could be considered as the high-level representation of residue co-evolution (Vig *et al.* 2021). In contrast, the residue pair-based features from other methods exhibited much more drastic decreases. For example, the AUCs of co-evolutionary features of PCTpred and PTM-X were reduced from 0.711 and 0.657 to 0.526 and 0.517, respectively (Supplementary Table S6). Collectively, our descriptors generally have less distance dependencies, and DeepPCT could be more robust in different predictive scenarios.

### 3.4 DeepPCT outperforms previous methods

To further demonstrate the advantages of our approach, we compared DeepPCT with existing machine learning-based methods. Note that we retrained the competing methods using the AlphaFold2-based structures of our training set and applied them to the newly collected testing set. As shown in Supplementary Tables S7–S10, DeepPCT yielded optimal performance for both sample- and protein-based evaluations on the training set, followed by PCTpred and PTM-X. For the testing set, our algorithm surpassed PCTpred and PTM-X by 0.082 and 0.095 in AUC and 0.075 and 0.088 in AUPR, respectively (Table 2). As discussed previously, if a model is highly dependent on the distance between PTMs, its generalizability could become weak. All the five features of PTM-X exhibited a strong distance dependency, but the distance difference between the positive and negative samples of the testing set (sequence distance: *P* = 1.66e−2) was much smaller than that of the training set (sequence distance: *P* = 4.43e−59). This led to the worst performance generated by PTM-X. In contrast, DeepPCT and PCTpred, which were less dependent on the distance measure, tended to perform more stably on new data. Besides, DeepPCT may also benefit from the protein-based optimization strategy. Previous methods relied on the sample-based optimization and lacked a rigorous independent testing, thus having the potential issue of overfitting. We still observed the complementarity between the three classifiers of DeepPCT on testing samples. The combination of DeepPCTsite and DeepPCTgraph induced an increment of approximately 0.03 in AUC, while the integration of DeepPCTstr and DeepPCTseq resulted in an increase of approximately 0.01. However, this tendency was not observed for other methods. For instance, PTM-X performed even worse than PTM-Xseq on testing data (AUC: 0.667 versus 0.688). This is because the difference in structural distance between positive and negative pairs was less significant (*P* = 0.466). Therefore, PTM-Xstr, which only adopted the structural distance as input, achieved very poor results (AUC: 0.543), and incorporation of this feature exerted a negative effect. This further suggests the importance of reducing distance dependency.

**Table 2.** Comparison between DeepPCT and existing methods on testing set.

Method	AUC	AUPR	MCC	F1
DeepPCTsite	0.684	0.130	0.129	0.171
DeepPCTsite^AF3^	0.754	0.173	0.175	0.193
DeepPCTgraph	0.722	0.106	0.127	0.169
DeepPCTgraph^AF3^	0.735	0.186	0.175	0.194
DeepPCTstr	0.754	0.127	0.156	0.197
DeepPCTstr^AF3^	0.765	0.245	0.220	0.242
DeepPCTseq	0.733	0.238	0.209	0.167
DeepPCT	0.762	0.240	0.210	0.235
DeepPCT^AF3^	0.777	0.278	0.245	0.258
PCTseq	0.664	0.182	0.123	0.168
PCTstr	0.676	0.137	0.132	0.172
PCTstr^AF3^	0.655	0.125	0.152	0.191
PCTpred	0.680	0.165	0.154	0.195
PCTpred ^AF3^	0.678	0.151	0.161	0.202
PTM-Xseq	0.688	0.166	0.233	0.252
PTM-Xstr	0.543	0.075	0.106	0.151
PTM-Xstr ^AF3^	0.528	0.112	0.105	0.151
PTM-X	0.667	0.152	0.122	0.166
PTM-X ^AF3^	0.671	0.156	0.126	0.167

AF3 denotes that AlphaFold3-based predicted structures were used.

PCTseq and PCTstr are the sequence and structure-based predictors of PCTpred, respectively.

PTM-Xseq and PTM-Xstr denote only using sequence and structural features of PTM-X, respectively.

As shown in Table 2, the individual and integrated modules of DeepPCT were superior to the corresponding parts of PCTpred. Thus, we summarize the advantage of current method over our previous algorithm (Supplementary Fig. S4). Regarding the sequence-based module, the residue and residue pair embeddings generated by PLMs were utilized to substitute the hand-crafted residue- and residue pair-based features. Meanwhile, the prediction engine was updated from a random forest model to a deep learning model, which used the attention mechanism to effectively extract residue representations from sequence windows. Regarding the structure-based module, we designed not only a machine learning predictor but also a deep learning predictor. Following the design concept of DeepPCTseq, we adopted the structural embedding of spatial windows of PTM pairs as the input of a graph neural network to implement DeepPCTgraph. In the machine learning component (DeepPCTsite), we retained the framework of PCTstr but used a group of novel hand-crafted features and reduced residue pair-based features to mitigate the distance dependency. Regarding the ensemble module, our current algorithm was the mixed combination of two deep learning predictors and one machine learning predictor, whereas our previous method was the pure integration of two machine learning predictors. Additionally, DeepPCT had higher computational efficiency than PCTpred. The inference time of DeepPCT for the testing set was approximately 10 minutes, compared to 10 hours for PCTpred (tested on Intel Xeon Silver 4116 T CPU).

When we are preparing this manuscript, AlphaFold3 was released with extended capabilities and improved performances (Abramson *et al.* 2024). We therefore re-evaluated our method using AlphaFold3-based structures. As shown in Tables 1 and 2, the performance of the structure-based predictors and the finally integrated model was slightly improved on the training and testing sets. Besides, certain modules achieved a marked increase in specific measures on testing samples (e.g. AUC: 0.684 versus 0.754 for DeepPCTsite). By comparing the structural similarities between native and predicted structures of 59 proteins in our datasets using the TM-align program (Zhang and Skolnick 2005), we found that AlphaFold3-based structures were closer to native structures than AlphaFold2-based counterparts (average TM-score: 0.763 versus 0.756). Thus, the increase in performance could be attributed to the improved accuracy of predicted structures. Collectively, as the quality of predicted structures increases, the utility of DeepPCT could be further enhanced.

## 4 Discussion

Although machine learning-based methods have been developed to predict PTM crosstalk within proteins, the prediction performance remains to be further improved. Application of deep learning to this field would be an emerging and promising way to solve this problem. Besides, the availability of high-quality predicted structures could facilitate the development of prediction models. Accordingly, we proposed DeepPCT, which is a deep learning framework to identify the interplay between PTMs based on AlphaFold2-based predicted structures. Our algorithm comprised two deep learning classifiers for sequence- and structure-based predictions, respectively, both of which took the embedding vector derived from pre-trained models as inputs. Meanwhile, a group of hand-crafted descriptors were newly applied to establish another structure-based classifier. Comprehensive evaluations indicate that the three classifiers offered complementary information and the ensemble model can yield the best measures for both sample- and protein-based assessments. The usage of predicted structures in this work implies that the application range of our method may be broad. Compared with existing methods, DeepPCT had better performance in different scenarios, especially showing strong generalizability on newly collected data.

Despite these progresses achieved here, several problems are worthy of studying in the future work. First, we only used the original structures to develop our model and neglected the information of structures with modified sites. Recent advances (e.g. RoseTTAFold All-Atom and AlphaFold3) enable the prediction of protein structures with PTMs (Abramson *et al.* 2024, Krishna *et al.* 2024). Therefore, the modified structures could be adopted to extract indicators of crosstalk events. Second, although the deep learning and embedding features had clearly improved the performance, the interpretability became weak compared to that of machine learning and traditional features. The learned patterns need to be further elucidated. Third, the present algorithm could only evaluate whether a PTM pair is involved in crosstalk events. As more suitable data become available, we will develop models to predict the interaction type of associated PTM sites (i.e. facilitate, inhibit, and co-operate) (Huang *et al.* 2015). Fourth, due to the limitation of existing experimental techniques, our knowledge of proteome-wide crosstalk events is very limited. Leveraging the inference efficiency of our algorithm, we could apply DeepPCT to all human protein structures in the AlphaFold database, which would provide an alternative avenue for investigating the proteome landscape and structural patterns of PTM crosstalk events. In summary, DeepPCT is an effective tool to explore the relationship between PTMs, which could advance our knowledge of crosstalk events in proteins.

## Supplementary Material

btae675_Supplementary_Data
