# Supplementary material for: Improved prediction of post-translational modification crosstalk within proteins using DeepPCT
Source: Bioinformatics. 2024 Nov 21;40(12):btae675. doi: 10.1093/bioinformatics/btae675 (PMC11645436; doi:10.1093/bioinformatics/btae675)
Supplement: btae675_Supplementary_Data [file btae675_supplementary_data.pdf]

Supplementary materials

Improved prediction of post-translational modification crosstalk within proteins using DeepPCT

Yu-Xiang Huang and Rong Liu*

Hubei Key Laboratory of Agricultural Bioinformatics, College of Informatics, Huazhong Agricultural University, Wuhan, 430070, P. R. China.

*Corresponding author

Email: liurong116@mail.hzau.edu.cn

Tel: +86-27-87280877

Fax: +86-27-87280877

Running title: Deep learning-based prediction of PTM crosstalk

Text S1. Construction of Set-random, Seq-control and Str-control datasets

To construct these three datasets, we selected all the positive samples and an equal number of negative samples from the training set. For the Set-random, the negative samples were randomly chosen. For the Seq-control and Str-control, the positive and negative samples were divided into bins based on the sequence distance (in steps of five residues) and structural distance (in steps of 5 Å, measured between C_α atoms), respectively. From each bin, we extracted all the positive samples and randomly selected an equal number of negative samples to ensure that the distributions of positive and negative samples were similar. Considering the down-sampling involved in these procedures, each sampling process was repeated 100 times. The average results of 100 repetitions were reported.

Text S2. Comparison of embeddings from different pre-trained models

Because our approach adopted residue pair embeddings to characterize the association between PTM sites, several factors need to be considered when selecting a PLM for generating sequence embeddings. First, we excluded PLMs without self-attention operations (e.g., ProteinBERT), because they cannot generate the necessary attention scores to constitute residue pair embeddings (Brandes, *et al.*, 2022). Second, we excluded PLMs having restrictions on sequence length (e.g., ESM-1b), because they divide the long sequence into chunks and thus prevent the generation of attention scores for PTM sites from different chunks (Rives, *et al.*, 2021). Third, PLMs without bidirectional attention (e.g., ProtGPT2) are less suitable, because these models cannot capture the dependencies between PTM sites in both directions and the residue embedding depends mainly on the preceding sequence rather than the entire context (Ferruz, *et al.*, 2022). Considering the above issues, we chose several suitable PLMs, including ESM-2, TAPE and ProtBert and ProtT5-XL from the ProtTrans series (Elnaggar, *et al.*, 2021; Rao, *et al.*, 2019). Regarding the structural embedding, we selected GearNet-Edge and ESM-IF (Hsu, *et al.*, 2022). As shown in Supplementary Table S2, ESM-2 and GearNet-Edge performed most favorably among sequence and structural models, respectively.

Text S3. Detailed information about structural descriptors

(1) Geometry-based descriptors

We adopted the circular variance (CV) to measure the density of atoms surrounding a residue (Ceres, *et al.*, 2012). A higher CV value indicates a higher density around the residue, suggesting that this residue may be deeply buried within the protein. For a given atom i, $\mathrm{CV}(i)$ is calculated as follows:

$$\mathrm{CV}(i) = 1 - \frac{1}{n_i}\left|\sum_{j \neq i, r_j \leq r_c} \frac{r_{ij}}{|r_{ij}|}\right|$$

where n_i denotes the number of atoms within a sphere of radius r_c (e.g., 10 Å) centered on atom i, and $r_{ij}/|r_{ij}|$ is the unit vector from i to j. The CV measure of a residue was computed by averaging the CV values of its atoms.

The Osipov-Pickup-Dunmur (OPD) chirality index was adopted to evaluate the conformational chirality of the local context of a given residue (Millar, *et al.*, 2005; Osipov, *et al.*, 1995). Non-chiral regions in proteins are expected to exhibit zero values, whereas chiral regions could have positive or negative values. For instance, the helical conformations in natural proteins are generally right-handed, possessing the positive OPD values. By contrast, the coil and sheet regions commonly have smaller or negative OPD values (Supplementary Figure S2). Accordingly, this descriptor could serve as an indicator of secondary structure. To calculate OPD chirality index, we selected the target residue along with its $(N-1)$-nearest neighbor residues based on the structural distance between C_α atoms. The formula can be presented as follows:

$$\mathrm{OPD}(N) = \frac{1}{3}\frac{4!}{N^4}\left[\sum_{\text{all permunation of } 1\ldots N} w_i w_j w_k w_l \times \frac{\left[(r_{ij} \times r_{kl}) \cdot r_{il}\right](r_{ij} \cdot r_{jk})(r_{jk} \cdot r_{kl})}{\left(|r_{ij}| \cdot |r_{jk}| \cdot |r_{kl}|\right)^2 \cdot r_{il}}\right]$$

where w_x denotes the molecular weight of the xth residue, and r_{xy} denotes the vector from C_α atom

of the xth residue to that of the yth residue. As suggested by Cha *et al*'s work, we used $N = 5, 7, 10, 15$ to calculate OPD chirality index values (Cha*, et al.*, 2022).

Additionally, three local descriptors, namely the accessible shell volume, minimum inaccessible radius, and pocketness, were used to quantify the depth and exposure of a given residue from different perspectives (Cha*, et al.*, 2022; Kawabata, 2010; Kawabata, 2019; Kawabata and Go, 2007). A higher accessible shell volume or minimum inaccessible radius indicates the residue is located at a protruding partition of the protein surface, while a lower pocketness value suggests the residue is presented at the shallow region of a protein pocket. These descriptors were generated using a pocket identification program called GHECOM (Kawabata, 2010).

(2) Graph-based descriptors

Each protein could be transformed into a graph, where nodes denote residues, and edges were formed between two residues if the distance between their C_α atoms was less than 7 Å. Based on this graph, the shortest path distance was calculated for two PTM sites and used as a residue pair-based descriptor to quantify their spatial proximity (Liu and Liu, 2020).

Moreover, the Ollivier Ricci curvature (ORC) was utilized to delineate areas within protein structures that are susceptible to conformational changes (Cha*, et al.*, 2022; Sia*, et al.*, 2019). ORC is based on the optimal transportation theory, and can be computed as follows:

$$ORC(x) = \sum_{y \in e(x,y)} ORC_e(x,y) = \sum_{y \in e(x,y)} 1 - \frac{W(m_x, m_y)}{d(x,y)}$$

where $e(x,y)$ denotes the set of edges connecting nodes x and y, m_x and m_y represent the density distributions of these nodes, and $W(m_x, m_y)$ is the Wasserstein distance, which quantifies the minimal cost of converting m_x into m_y. Edge-based curvatures (i.e., $ORC_e(x,y)$) was yielded by the GraphRicciCurvature package (Ni*, et al.*, 2019).

We also employed the node-based multifractal dimension (MFD), which is an indicator to measure the network complexity in multifractal analysis (Cha*, et al.*, 2022; Xue and Bogdan, 2017). A higher value indicates a higher degree of complexity. Through the box-covering method, we evaluated the scaling relationship between the mass distribution within a box and the box size centered on a given node. The relationship could be presented as follows:

$$y = \ln\left(\frac{M(l)}{l}\right)$$

where l represents the maximum shortest path distance from the target node to any other node within the box, and $M(l)$ is the number of nodes within the box, indicating the mass distribution.

Text S4 Construction of baselines for the three basic classifiers

(1) Baselines for DeepPCTseq

To establish baselines models, we used convolutional neural network (CNN) and long short-term memory network (LSTM) architectures to substitute the cross-attention layers in DeepPCTseq. For the CNN baseline, the two sequence windows, $X^{(i)}$ and $X^{(j)}$, were concatenated into a larger matrix H_1. A convolutional operation was then applied to H_1 using a 2D convolutional kernel of size 3×3, followed by a 2×2 max pooling operation to extract features as follows:

$$H_2 = \mathrm{MaxPool}\left(\mathrm{ReLU}\left(\mathrm{Conv2D}(H_1)\right)\right)$$

where the resulting H_2 was flattened into a vector H_3. This vector was concatenated with the residue pair embedding $X^{(ij)}$ to form an extended vector H_4, which was then fed into a fully connected network to produce output probability.

For the LSTM baseline, we utilized an LSTM layer to process the combined sequence window H_1. The matrix H_1 consists of 22 vectors, each of which has a dimension of 1280. The LSTM layer has a hidden size of 640. Each vector in H_1 was sequentially input to the LSTM unit, which used the current input vector x_t, the previous hidden state h_{t-1}, and the previous cell state c_{t-1} to compute the new hidden state h_t and cell state c_t as follows:

$$h_t, c_t = \mathrm{LSTMUnit}(x_t, h_{t-1}, c_{t-1})$$

where we obtained the final hidden state h_n from the last step, which represents the aggregated information of the entire sequence. h_n was concatenated with the residue pair embedding $X^{(ij)}$ to form an extended vector, which was fed into a fully connected network to yield output probability.

(2) Baselines for DeepPCTgraph

To construct the graph-based baseline models, we replaced the graph isomorphism network in DeepPCTgraph with the graph convolutional network (GCN), graph attention network (GAT), and

GraphSAGE to update node features (Hamilton, *et al.*, 2017; Kipf and Welling, 2016; Veličković, *et al.*, 2018). The input and output dimensions of each layer were identical for different models. For the GAT baseline, the number of attention heads was three, and the updated node features were averaged across these heads. For the GraphSAGE baseline, the mean aggregator was used. Afterwards, all three baseline models employed the same readout operation and linear layer of DeepPCTgraph to obtain graph representations and generate output probabilities.

For the CNN and LSTM baselines, the input node features were processed as descripted in 'Baselines for DeepPCTseq' section. For each graph, the feature vectors derived from the 22 nodes formed a matrix X_G. The CNN model included a 2D convolutional layer with a 3×3 kernel, followed by a ReLU activation function and a 2×2 max pooling operator. The resulting features were flattened and passed through a fully connected network to produce outputs. In the LSTM baseline, the 22 vectors with a dimension of 3072 from X_G were sequentially fed into an LSTM unit with a hidden size of 1536. The final hidden state was passed through a fully connected network to yield outputs.

(3) Baselines for DeepPCTsite

We established baseline models through five widely used machine learning algorithms, including the support vector machine (SVM), extreme gradient boosting (XGBoost), logistic regression (LR), neural network (NN), and naive Bayes (NB). We optimized their parameters based on the training set. For SVM, the radial basis function was adopted as a kernel, and the parameters C and γ were set to 0.5 and 1, respectively. For XGBoost, we assigned the maximum depth to 8 and the learning rate to 0.2. For LR, we adopted an L_2 penalty with the parameter C of 2. For NN, the hidden layer size was set to 15 based on a ReLU activation function. For NB, we used the Gaussian algorithm with the variance smoothing parameter of 1e-10.

Text S5. Different methods for integrating sequence and structural information

In this study, each sample was characterized by three types of features: residue and residue pair embeddings, structural embeddings, and structural descriptors. These features were processed by individual classifiers, and their output probabilities were merged using a weighted sum method. In fact, we attempted to design a single deep learning framework, which took the three types of features as input to yield a prediction score. As shown in Supplementary Table S5, however, these attempts were inferior to the weighted combination method. We briefly introduce the alternative integrative methods as follows:

Method 1

We obtained the updated representation of residue and residue pair embeddings using the DeepPCTseq module without a fully connected layer. Similarly, the updated representation of structural embeddings was generated by the DeepPCTgraph module. The hand-crafted structural descriptors were input into a multi-layer perceptron module to yield an updated representation. The representations produced by the three modules were concatenated and fed into a fully connected network to generate the prediction score.

Method 2

The updated representation of residue and residue pair embeddings and that of structural embeddings were generated as suggested in Method 1. These two types of representations combined with the raw structural descriptors were input into a fully connected network to produce the prediction score.

Method 3

Inspired by the mixture of experts (MoE) technique, we tried to combine different modules with

dynamic weights (Shazeer, *et al.*, 2017). The original DeepPCTseq and DeepPCTgraph modules were adopted, while a multi-layer perceptron module was constructed to process the structural descriptors. For each sample, different types of features were fed into the corresponding modules to obtain crosstalk probabilities as follows:

$$P_{\text{seq}} = \text{DeepPCTseq}\left(x^{(i)}, x^{(j)}, x^{(ij)}\right)$$

$$P_{\text{graph}} = \text{DeepPCTgraph}(\mathcal{G})$$

$$P_{\text{MLP}} = \text{MLP}(\text{structural descriptors})$$

where $x^{(i)}$ and $x^{(j)}$ denote the sequence windows, $x^{(ij)}$ represents the residue pair embedding, and $\mathcal{G}$ is the graph of a spatial microenvironment. Additionally, a gating network was built to assign the weights of three modules for each sample. This network took all the features as input and predicted the optimal weights as follows:

$$w_1, w_2, w_3 = \text{Softmax}\left(\text{GatingNetwork}\left(x^{(i)}, x^{(j)}, x^{(ij)}, \mathcal{G}, \text{structural descriptors}\right)\right)$$

where w_1, w_2 and w_3 are the assigned weights for three modules. Finally, the prediction score (P) was calculated as follows:

$$P = w_1 P_{\text{seq}} + w_2 P_{\text{graph}} + w_3 P_{\text{MLP}}$$

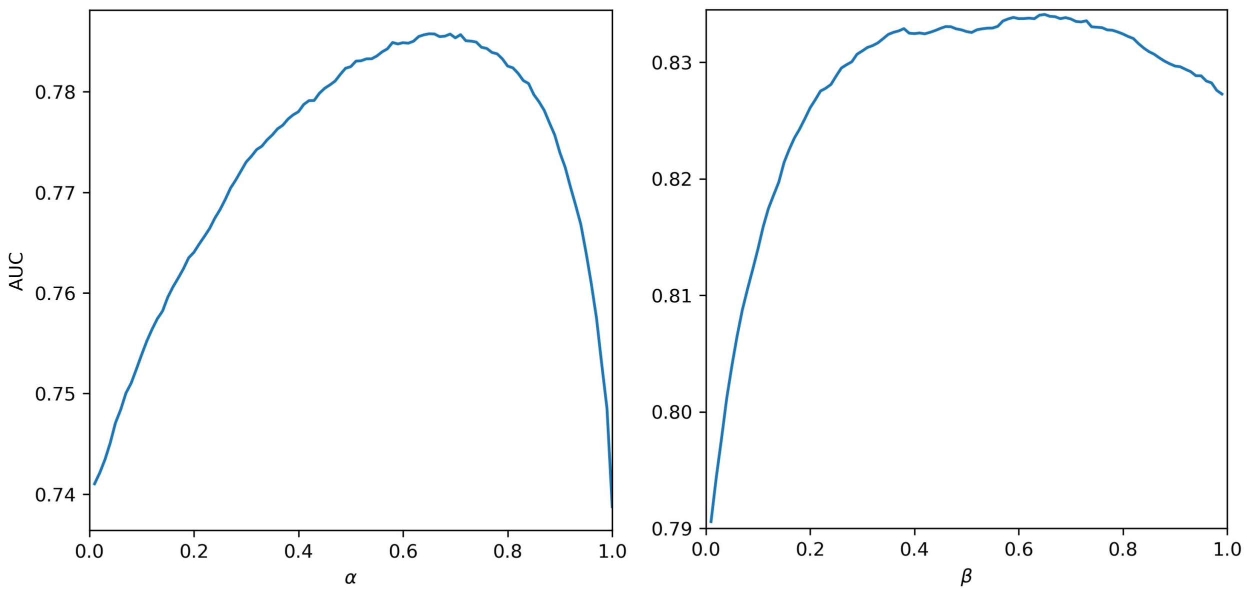

Figure S1. Selection of α and β in the integrated model. These two parameters are determined by adjusting them with a step size of 0.01 and observing the AUC measures on the training set. The AUC reaches its peak when both α and β are set to 0.65.

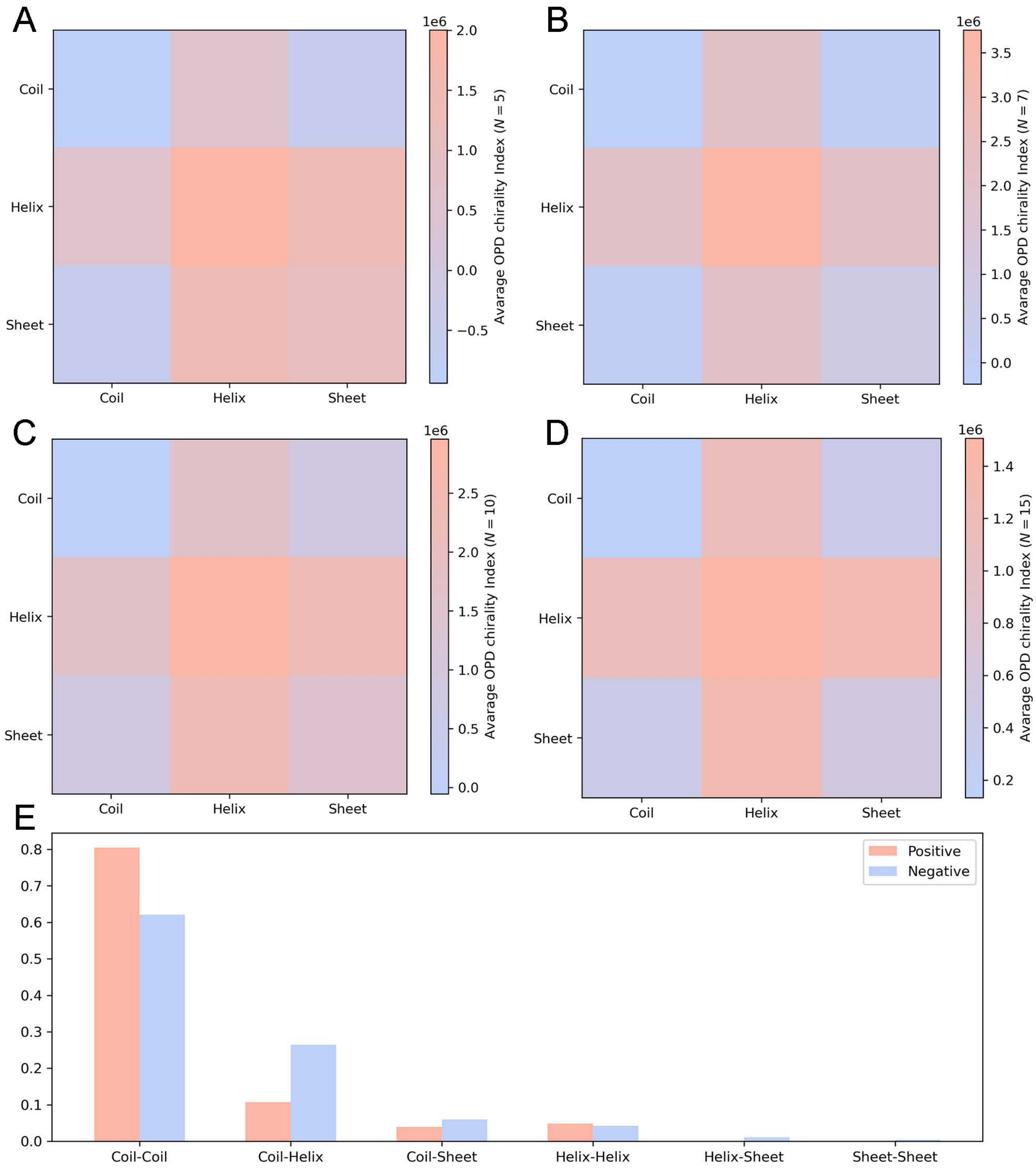

Figure S2. Analysis of OPD chirality indices. (A-D) Average chirality index of samples having different secondary structure combinations. Each figure corresponds to a different N value for computing the index. Residues annotated as H, G, and I by the DSSP program are considered to be in the helical conformation, residues annotated as E and B are in the sheet conformation, and the remaining residues are in the coil conformation. (E) Proportion of different secondary structure combinations for positive and negative samples.

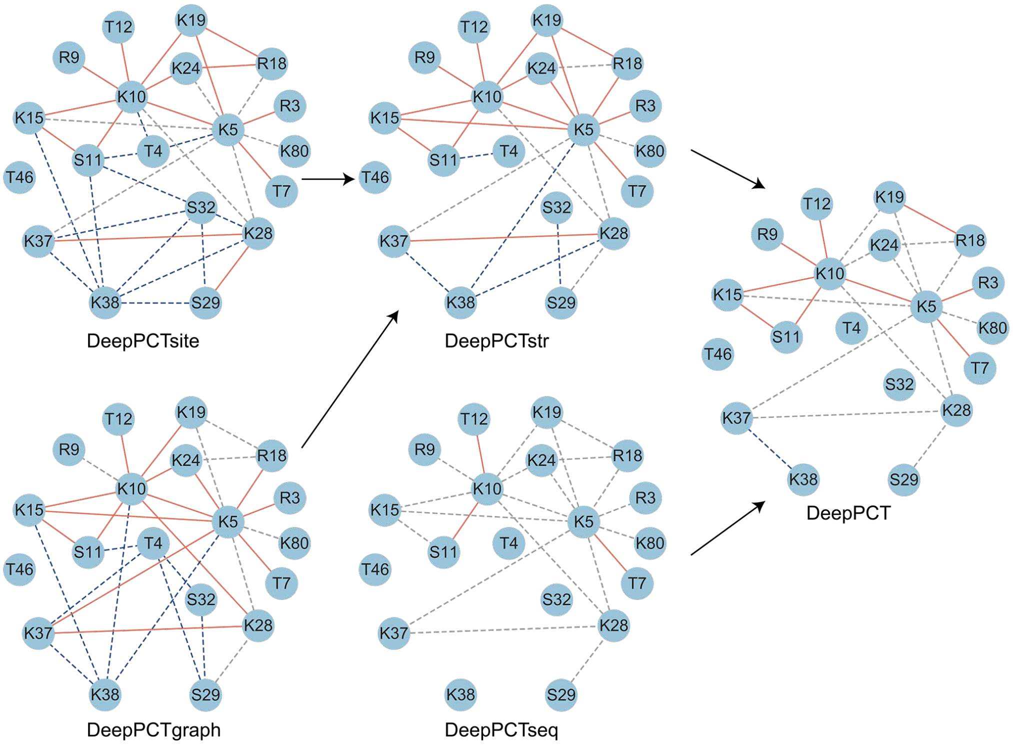

Figure S3. Visualization of prediction results of PTM pairs in histone H3 using protein-based evaluation. True positives, false positives, and false negatives are highlighted in red, blue, and gray, respectively.

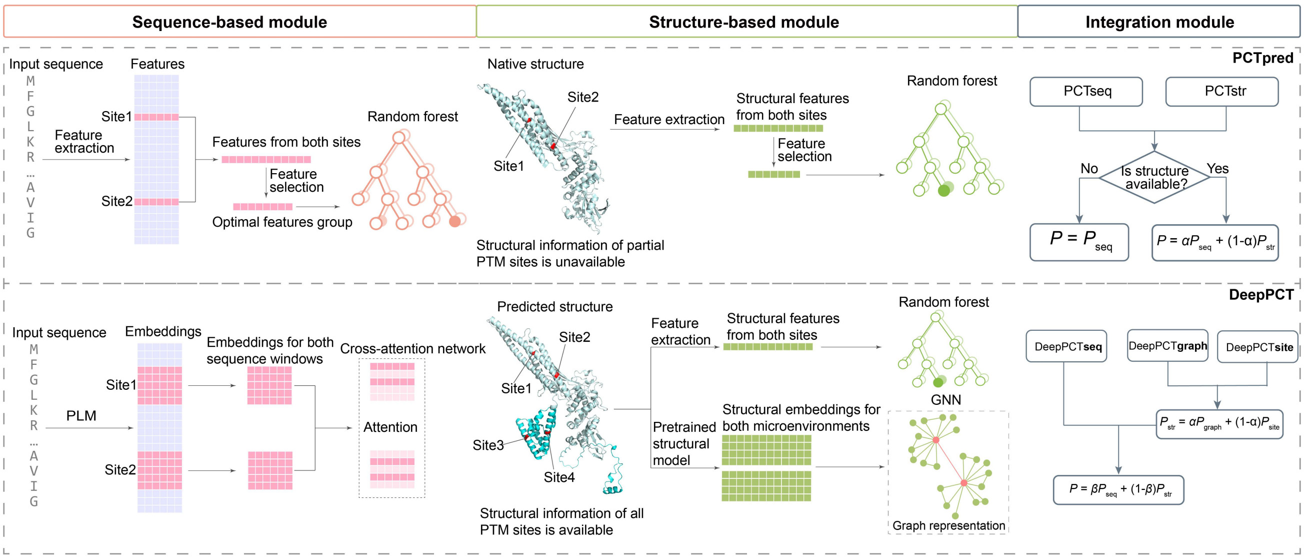

Figure S4. Systematic comparison of DeepPCT and PCTpred. A detailed description is provided in the second paragraph of 'DeepPCT outperforms previous methods' section in the main text.

Table S1. Experimentally validated PTM crosstalk pairs collected from literatures

Protein name	UniProt ID	Site1	PTM1	Site2	PTM2	Relationship	PubMed ID
MCL1	Q07820	K40	Acetylation	S159	Phosphorylation	inhibit	34758305
MCL1	Q07820	K40	Acetylation	T163	Phosphorylation	inhibit	34758305
RNF113A	O15541	K20	Methylation	S6	Phosphorylation	facilitate	35819319
RNF114A	O15541	K20	Methylation	S43	Phosphorylation	facilitate	35819319
RNF115A	O15541	K20	Methylation	S45	Phosphorylation	facilitate	35819319
RNF116A	O15541	K20	Methylation	S46	Phosphorylation	facilitate	35819319
RNF117A	O15541	K20	Methylation	S47	Phosphorylation	facilitate	35819319
Fascin	Q16658	S32	Phosphorylation	K471	Acetylation	co-operate	35514194
Sox2	P48431	S251	Phosphorylation	K245	SUMOylation	facilitate	29556337
Sox10	P56693	T240	Phosphorylation	K55	SUMOylation	inhibit	29295999
Sox10	P56693	T244	Phosphorylation	K55	SUMOylation	inhibit	29295999
Src	P12931	Y419	Phosphorylation	K318	SUMOylation	inhibit	29069627
FNIP1	Q8TF40	S938	Phosphorylation	S939	Phosphorylation	facilitate	30699359
FNIP1	Q8TF40	S939	Phosphorylation	S941	Phosphorylation	facilitate	30699359
FNIP1	Q8TF40	S941	Phosphorylation	S946	Phosphorylation	facilitate	30699359
FNIP1	Q8TF40	S946	Phosphorylation	S948	Phosphorylation	facilitate	30699359
FNIP1	Q8TF40	S938	O-GlcNAcylation	K1119	Ubiquitination	facilitate	30699359
CDCP1	Q9H5V8	Y734	Phosphorylation	Y707	Phosphorylation	facilitate	31606391
CDCP1	Q9H5V8	Y734	Phosphorylation	Y806	Phosphorylation	facilitate	31606391
ZO-3	O95049	Y364	Phosphorylation	S369	O-GlcNAcylation	inhibit	30725225

The Src protein has the highest sequence identity relative to proteins in the training set (53.5%), while other proteins in this dataset have a value from 3.2% to 37.7%.

Table S2. Performance of embeddings from different pre-trained models on training set

Classifier	Pre-trained model	AUC	AUPR	MCC	F1	Recall	Precision	ACC
DeepPCTseq	ESM-2	0.827	0.368	0.318	0.271	0.218	0.648	0.949
	ProtT5-XL	0.821	0.333	0.297	0.280	0.224	0.495	0.944
	ProtBert	0.804	0.260	0.267	0.263	0.258	0.381	0.945
	TAPE	0.743	0.253	0.234	0.174	0.109	0.567	0.942
DeepPCTgraph	GearNet-Edge	0.739	0.182	0.167	0.183	0.188	0.289	0.942
	ESM-IF	0.658	0.133	0.112	0.132	0.464	0.090	0.791

Table S3. Dimensions of features used in each classifier

Classifier	Feature	Dimension
DeepPCTseq	Residue embedding	1280
	Residue pair embedding	1320
DeepPCTgraph	Structural embedding	3072
DeepPCTsite	Shortest path distance	1
	Circular variance	1
	Accessible shell volume	1
	Minimum inaccessible radius	1
	Pocketness	1
	Multifractal dimension	1
	OPD chirality index	4
	Ollivier-Ricci curvature	1

Table S4. Performance of baseline for the three basic classifiers on training set

Classifier	Baseline	AUC	AUPR	MCC	F1	Recall	Precision	ACC
DeepPCTseq	CNN	0.774	0.312	0.281	0.259	0.198	0.51	0.945
	LSTM	0.739	0.213	0.220	0.217	0.269	0.355	0.940
DeepPCTgraph	CNN	0.609	0.110	0.151	0.094	0.055	0.492	0.943
	LSTM	0.521	0.088	0.053	0.098	0.702	0.076	0.419
	GCN	0.700	0.140	0.135	0.147	0.131	0.240	0.939
	GAT	0.706	0.149	0.119	0.149	0.242	0.136	0.905
	GraphSAGE	0.683	0.179	0.151	0.107	0.064	0.405	0.943
DeepPCTsite	SVM	0.622	0.114	0.070	0.062	0.044	0.166	0.939
	XGBoost	0.717	0.144	0.122	0.127	0.093	0.239	0.941
	LR	0.719	0.143	0.156	0.164	0.126	0.277	0.944
	NN	0.705	0.126	0.129	0.144	0.548	0.100	0.767
	NB	0.586	0.114	0.081	0.124	0.279	0.106	0.841

Table S5. Performance of different integration methods on training set

Method	AUC	AUPR	MCC	F1	Recall	Precision	ACC
Method 1	0.766	0.243	0.293	0.248	0.202	0.623	0.967
Method 2	0.773	0.232	0.297	0.259	0.182	0.607	0.967
Method 3	0.820	0.340	0.298	0.287	0.522	0.271	0.915
DeepPCT	0.834	0.375	0.349	0.337	0.329	0.497	0.953

Table S6. Distance dependency of individual features used by existing methods

Method	Feature	Set-random (AUC)	Seq/Str-control (AUC)
PCTpred	Sequence distance	0.763	0.351
	Residue co-evolution	0.711	0.526
	Co-localization within the same disorder region	0.761	0.546
	Co-localization within the same domain	0.569	0.522
	Correlated mutation	0.767	0.674
	Residue conservation	0.666	0.645
	SIFT score	0.645	0.607
	Polyphen-2 score	0.656	0.616
	Structural distance	0.722	0.482
	Shortest path distance	0.732	0.500
	Co-localization within the same pocket	0.519	0.502
	Pairwise secondary structure state	0.591	0.607
	Topological features	0.790	0.750
	Laplacian norm	0.775	0.722
	Depth and protrusion indices	0.706	0.705
	pLDDT	0.650	0.630
	Number of hydrogen bonds	0.592	0.609
PTM-X	Sequence distance	0.763	0.351
	Residue co-evolution	0.657	0.517
	Modification co-evolution	0.621	0.595
	Disorder location	0.674	0.507
	Structural distance	0.722	0.482

PCTpred and PTM-X used the direct information and mutual information as residue co-evolutionary features, respectively.

Because the B-factor feature used by PCTpred is only available for native structures, we used a comparable feature, the predicted local distance difference test (pLDDT) score produced by AlphaFold2.

Table S7. Performance of different methods on training set using sample-based evaluation

Classifier	AUC	AUPR	MCC	F1	Recall	Precision	ACC
DeepPCTsite	0.894	0.302	0.338	0.280	0.180	0.668	0.985
DeepPCTsite^{AF3}	0.889	0.290	0.344	0.336	0.518	0.249	0.967
DeepPCTgraph	0.911	0.493	0.476	0.435	0.326	0.743	0.987
DeepPCTgraph^{AF3}	0.924	0.538	0.548	0.526	0.440	0.730	0.987
DeepPCTstr	0.942	0.525	0.503	0.471	0.371	0.733	0.987
DeepPCTstr^{AF3}	0.947	0.572	0.557	0.553	0.528	0.622	0.986
DeepPCTseq	0.946	0.643	0.596	0.587	0.504	0.728	0.989
DeepPCTseq^{nrp}	0.925	0.586	0.569	0.556	0.459	0.727	0.989
DeepPCT	0.957	0.663	0.620	0.616	0.552	0.716	0.989
DeepPCT^{AF3}	0.956	0.662	0.627	0.627	0.602	0.676	0.989
PCTseq	0.890	0.252	0.305	0.303	0.440	0.238	0.967
PCTstr	0.851	0.173	0.254	0.242	0.448	0.168	0.954
PCTstr^{AF3}	0.867	0.227	0.286	0.277	0.462	0.201	0.961
PCTpred	0.890	0.254	0.306	0.306	0.428	0.246	0.968
PCTpred	0.899	0.279	0.326	0.327	0.438	0.271	0.971
PTM-Xseq	0.814	0.121	0.221	0.207	0.432	0.138	0.946
PTM-Xstr	0.759	0.098	0.204	0.178	0.482	0.109	0.927
PTM-Xstr^{AF3}	0.767	0.109	0.204	0.176	0.493	0.108	0.924
PTM-X	0.822	0.135	0.235	0.200	0.548	0.123	0.928
PTM-X^{AF3}	0.824	0.133	0.230	0.196	0.533	0.121	0.929

AF3 denotes that AlphaFold3-based predicted structures were used.

DeepPCTseq^{nrp} denotes that the residue pair embedding was deleted in DeepPCTseq.

PCTseq and PCTstr are the sequence and structure-based predictors of PCTpred, respectively.

PTM-Xseq and PTM-Xstr denote only using sequence and structural features of PTM-X, respectively.

Table S8. Performance of different methods on training set using protein-based evaluation

Classifier	AUC	AUPR	MCC	F1	Recall	Precision	ACC
DeepPCTsite	0.736	0.149	0.138	0.115	0.511	0.108	0.816
DeepPCTsite^{AF3}	0.744	0.163	0.174	0.193	0.273	0.193	0.921
DeepPCTgraph	0.739	0.182	0.167	0.183	0.188	0.289	0.942
DeepPCTgraph^{AF3}	0.747	0.189	0.179	0.158	0.144	0.443	0.951
DeepPCTstr	0.786	0.217	0.211	0.228	0.271	0.252	0.944
DeepPCTstr^{AF3}	0.799	0.224	0.222	0.242	0.261	0.273	0.942
DeepPCTseq	0.827	0.368	0.318	0.271	0.218	0.648	0.949
DeepPCTseq^{nrp}	0.775	0.269	0.222	0.215	0.507	0.198	0.859
DeepPCT	0.834	0.375	0.349	0.337	0.329	0.497	0.953
DeepPCT^{AF3}	0.838	0.381	0.357	0.348	0.338	0.492	0.953
PCTseq	0.792	0.234	0.247	0.251	0.446	0.206	0.924
PCTstr	0.757	0.155	0.219	0.222	0.498	0.158	0.903
PCTstr^{AF3}	0.774	0.184	0.226	0.234	0.464	0.177	0.912
PCTpred	0.791	0.211	0.258	0.273	0.424	0.224	0.932
PCTpred^{AF3}	0.799	0.225	0.271	0.279	0.424	0.241	0.935
PTM-Xseq	0.780	0.175	0.196	0.219	0.513	0.146	0.880
PTM-Xstr	0.734	0.140	0.205	0.211	0.476	0.155	0.900
PTM-Xstr^{AF3}	0.756	0.154	0.215	0.214	0.499	0.161	0.899
PTM-X	0.784	0.183	0.216	0.245	0.313	0.229	0.934
PTM-X^{AF3}	0.790	0.186	0.223	0.221	0.588	0.147	0.882

AF3 denotes that AlphaFold3-based predicted structures were used.

DeepPCTseq^{nrp} denotes that the residue pair embedding was deleted in DeepPCTseq.

PCTseq and PCTstr are the sequence and structure-based predictors of PCTpred, respectively.

PTM-Xseq and PTM-Xstr denote only using sequence and structural features of PTM-X, respectively.

Table S9. Performance of different methods on testing set

Method	AUC	AUPR	MCC	F1	Recall	Precision	ACC
DeepPCTsite	0.684	0.130	0.129	0.171	0.300	0.120	0.870
DeepPCTsite^{AF3}	0.754	0.173	0.175	0.193	0.150	0.273	0.944
DeepPCTgraph	0.722	0.106	0.127	0.169	0.300	0.118	0.868
DeepPCTgraph^{AF3}	0.735	0.186	0.175	0.194	0.150	0.273	0.944
DeepPCTstr	0.754	0.127	0.156	0.197	0.300	0.146	0.890
DeepPCTstr^{AF3}	0.765	0.245	0.220	0.242	0.200	0.308	0.944
DeepPCTseq	0.733	0.238	0.209	0.167	0.100	0.500	0.955
DeepPCT	0.762	0.240	0.210	0.235	0.200	0.286	0.942
DeepPCT^{AF3}	0.777	0.278	0.245	0.258	0.200	0.364	0.949
PCTseq	0.664	0.182	0.123	0.168	0.254	0.126	0.887
PCTstr	0.676	0.137	0.132	0.172	0.327	0.117	0.858
PCTstr^{AF3}	0.655	0.125	0.152	0.191	0.327	0.136	0.875
PCTpred	0.680	0.165	0.154	0.195	0.285	0.149	0.894
PCTpred^{AF3}	0.678	0.151	0.161	0.202	0.289	0.156	0.897
PTM-Xseq	0.688	0.166	0.233	0.252	0.487	0.171	0.870
PTM-Xstr	0.543	0.075	0.106	0.151	0.295	0.102	0.852
PTM-Xstr^{AF3}	0.528	0.112	0.105	0.151	0.286	0.103	0.856
PTM-X	0.667	0.152	0.122	0.166	0.225	0.133	0.899
PTM-X^{AF3}	0.671	0.156	0.126	0.167	0.330	0.112	0.853

AF3 denotes that AlphaFold3-based predicted structures were used.

PCTseq and PCTstr are the sequence and structure-based predictors of PCTpred, respectively.

PTM-Xseq and PTM-Xstr denote only using sequence and structural features of PTM-X, respectively.

Table S10. P-values of performance difference between various modules and methods

	Competing models	Training set (SE)	Training set (PE)	Testing set
P-value of AUC	Site v.s. Str	1.20e-11	5.15e-10	2.85e-04
	Graph v.s. Str	3.19e-09	4.48e-08	5.16e-03
	Seq v.s. DeepPCT	3.47e-08	5.56e-07	3.78e-03
	Str v.s. DeepPCT	2.52e-06	5.64e-08	2.84e-02
	DeepPCT v.s. PCTpred	7.17e-11	3.55e-07	1.57e-04
	DeepPCT v.s. PTM-X	1.44e-09	2.12e-09	8.42e-06
P-value of AUPR	Site v.s. Str	2.75e-13	9.48e-09	5.32e-01
	Graph v.s. Str	8.68e-08	2.71e-06	5.83e-06
	Seq v.s. DeepPCT	2.58e-05	2.67e-06	1.28e-03
	Str v.s. DeepPCT	3.86e-11	9.58e-12	7.97e-06
	DeepPCT v.s. PCTpred	4.78e-13	1.48e-09	9.60e-04
	DeepPCT v.s. PTM-X	8.83e-16	6.90e-12	7.40e-04

SE: Sample-based evaluation, PE: Protein-based evaluation, Site: DeepPCTsite, Graph: DeepPCTgraph, Str: DeepPCTstr, and Seq: DeepPCTseq.

Reference

Brandes, N.*, et al.* ProteinBERT: a universal deep-learning model of protein sequence and function. *Bioinformatics* 2022;38(8):2102-2110.

Ceres, N., Pasi, M. and Lavery, R. A Protein Solvation Model Based on Residue Burial. *J Chem Theory Comput* 2012;8(6):2141-2144.

Cha, M.*, et al.* Unifying structural descriptors for biological and bioinspired nanoscale complexes. *Nat Comput Sci* 2022;2(4):243-252.

Elnaggar, A.*, et al.* ProtTrans: Towards Cracking the Language of Lifes Code Through Self-Supervised Deep Learning and High Performance Computing. *IEEE Transactions on Pattern Analysis and Machine Intelligence* 2021:1-1.

Ferruz, N., Schmidt, S. and Höcker, B. ProtGPT2 is a deep unsupervised language model for protein design. *Nature Communications* 2022;13(1):4348.

Hamilton, W.L., Ying, R. and Leskovec, J. Inductive representation learning on large graphs. In: *Advances in Neural Information Processing Systems*, 2017.

Hsu, C.*, et al.* Learning inverse folding from millions of predicted structures. In: *International Conference on Machine Learning*, 2022.

Kawabata, T. Detection of multiscale pockets on protein surfaces using mathematical morphology. *Proteins* 2010;78(5):1195-1211.

Kawabata, T. Detection of cave pockets in large molecules: Spaces into which internal probes can enter, but external probes from outside cannot. *Biophys Physicobiol* 2019;16:391-406.

Kawabata, T. and Go, N. Detection of pockets on protein surfaces using small and large probe spheres to find putative ligand binding sites. *Proteins* 2007;68(2):516-529.

Kipf, T.N. and Welling, M. Semi-Supervised Classification with Graph Convolutional Networks. In: *International Conference on Learning Representations*, 2016.

Liu, H.-F. and Liu, R. Structure-based prediction of post-translational modification cross-talk within proteins using complementary residue- and residue pair-based features. *Briefings in Bioinformatics* 2020;21(2):609-620.

Millar, G., Weinberg, N. and Mislow, K. On the Osipov–Pickup–Dunmur chirality index: why pseudoscalar functions are generally unsuitable to quantify chirality. *Molecular Physics* 2005;103(20):2769-2772.

Ni, C.-C.*, et al.* Community Detection on Networks with Ricci Flow. *Scientific Reports* 2019;9(1):9984.

Osipov, M.A., Pickup, B.T. and Dunmur, D.A. A new twist to molecular chirality: intrinsic chirality indices. *Molecular Physics* 1995;84(6):1193-1206.

Rao, R.*, et al.* Evaluating protein transfer learning with TAPE. In: *Advances in Neural Information Processing Systems*, 2019.

Rives, A.*, et al.* Biological structure and function emerge from scaling unsupervised learning to 250 million protein sequences. *Proc Natl Acad Sci U S A* 2021;118(15).

Shazeer, N.*, et al.* Outrageously Large Neural Networks: The Sparsely-Gated Mixture-of-Experts Layer. In: *International Conference on Learning Representations*, 2017.

Sia, J., Jonckheere, E. and Bogdan, P. Ollivier-Ricci Curvature-Based Method to Community Detection in Complex Networks. *Sci Rep* 2019;9(1):9800.

Veličković, P.*, et al.* Graph Attention Networks. In: *International Conference on Learning Representations*, 2018.

Xue, Y. and Bogdan, P. Reliable Multi-Fractal Characterization of Weighted Complex Networks: Algorithms and Implications. *Scientific Reports* 2017;7(1):7487.
